# Synergistic Anti-Inflammatory Effects of Ethanol Extracts from *Chrysanthemum zawadskii* Flower and *Cudrania tricuspidata* Fruit Occur via Inhibition of the NF-*κ*B Signaling Pathway

**DOI:** 10.1155/2023/8198228

**Published:** 2023-09-22

**Authors:** Jisun Hwang, Bohee Jang, Yeong Woo Choi, Inn-Oc Han, Eok-Soo Oh

**Affiliations:** ^1^Department of Life Sciences, Ewha Womans University, Seoul 03760, Republic of Korea; ^2^Department of Physiology and Biophysics, College of Medicine, Inha University, Incheon 22212, Republic of Korea

## Abstract

*Chrysanthemum zawadskii* (CZ) and *Cudrania tricuspidata* (CT) are both traditional Korea herbal medicines, which is widely used to treat fever, cough, gastritis, and women's diseases that may be linked to inflammatory response. Although it has been used to treat diseases related to inflammation, there has been no case of the synergistic anti-inflammatory properties of both extracts. Our data revealed that ethanol extracts of dried whole CZ exhibited free radical-scavenging capacity *in vitro*, reduced LPS-induced intracellular reactive oxygen species, and decreased the LPS-induced upregulations of the mRNAs encoding iNOS, COX-2, and IL-6 in RAW 264.7 cells, without significant cytotoxicity. This anti-inflammatory effect was most evident from flower extracts: ethanol extracts from flowers significantly reduced the LPS-induced upregulations of iNOS and COX-2 at a concentration of 100 *μ*g/ml. An ethanol extract of the fruit from CT also exerted a radical scavenging capacity and suppressed LPS-induced proinflammatory gene expression: 5.5 *μ*g/ml of the ethanol extract significantly reduced the ability of LPS to induce the mRNA expression levels of iNOS and IL-6 without apparent cytotoxicity. Furthermore, as little as 1.0 *μ*g/ml of the combined ethanol extracts of CZ flower and CT fruit reduced the LPS-induced changes monitored herein, decreasing the upregulations of iNOS and IL-6, and decreasing the nuclear localization of NF-*κ*B p65. These results suggest that the observed synergistic anti-inflammatory effects may be mediated via inhibition of NF-*κ*B signaling. Taken together, these data suggest that ethanol extracts from CZ flowers and CT fruits have synergistic anti-inflammatory effects and that a combination of the two extracts could prove useful for the treatment of inflammation-related diseases.

## 1. Introduction

Inflammation is part of the complex biological response that a body uses to mitigate injury or harmful stimuli, such as pathogens, damaged cells, or irritation. During the inflammatory response, various cells (known as inflammatory cells) release specialized substances, including vasoactive amines, vasoactive peptides, and proinflammatory cytokines. These inflammatory mediators are beneficial for host defense [1], but their excessive and chronic productions are also related to various diseases, such as diabetes, cancer, and infectious diseases [2]. For example, inflammation can lead to increases in reactive oxygen species (ROS) and free radicals, which damage cell structures, including DNA and surrounding tissues. Hence, natural active ingredients that act as antioxidants by scavenging free radicals and/or inhibiting the production of intracellular ROS (e.g., polyphenols, vitamins, and minerals) can prevent disease and reduce inflammation. Recently, a great deal of research effort has been devoted towards discovering anti-inflammatory compounds of plant origin as potential natural and safe medicines with no harmful side effects.

A variety of plants have traditionally been used in human medicine in Korea, and studies have shown that several Korean herbal medicines have anti-inflammatory effects. For instance, water extracts from *Stauntonia hexaphylla* fruit [3], ginseng root [4], and *Vaccinium oldhamii* leaves [5] are known to suppress the ability of lipopolysaccharide (LPS, an inflammatory stimulator) to induce the production of proinflammatory cytokines and mediators.

*Chrysanthemum zawadskii* (CZ) is a traditional Korean medicinal plant used to treat fever, cough, gastritis, and women's diseases that may be linked to inflammatory response. Several reports have shown that CZ extracts have anti-inflammatory effects. For example, Han et al. reported that linarin, a major physiological active compound from CZ, inhibited the LPS-induced productions of nitric oxide (NO) and cytokines in RAW 264.7 cells [6]. Methanol extracts from dried CZ leaf suppressed LPS-induced inducible nitric oxide synthase (iNOS) expression and NO production [7], and hexane and ethanol extracts of whole CZ plants inhibited LPS-induced interleukin- (IL-) 1*β* and cyclooxygenase- (COX-) 2 gene expression, and NO production [8].

Although nonsteroidal anti-inflammatory drugs (NSAIDs) are well known to reduce pain and exert excellent anti-inflammatory effects, they can cause multiple adverse effects [9], including gastrointestinal, cardiovascular, and renal complications [10]. Therefore, researchers are urgently seeking natural products that show anti-inflammatory effects and/or increase anti-inflammatory effects and without side effects. We herein investigated the anti-inflammatory effects of CZ extracts and their potential synergistic anti-inflammatory effects with extracts from *Cudrania tricuspidata* (CT), which is another Korean natural product.

## 2. Materials and Methods

### 2.1. Reagents

2,2′-Diphenyl-picryl-hydrazyl free radical (DPPH), 2,2′-azinobis-(3-ethylbenzothiazoline-6-sulphonic acid) (ABTS), lipopolysaccharide (LPS), dimethyl sulfoxide (DMSO), 2′,7′-dichlorofluorescein diacetate (DCF-DA), and 3-(4,5-dimethylthiazol-2-yl)-2,5-diphenyltetrazolium bromide (MTT) were purchased from Sigma-Aldrich (USA). The antibody against NF-*κ*B subunit p65 was purchased from Merck (Darmstadt, Germany).

### 2.2. Preparation of Extracts

*Chrysanthemum zawadskii* (CZ) and *Cudrania tricuspidata* (CT) fruit harvested in 2019 were purchased from a local store (j-dream, Jeongeup, South Korea). Before extraction, whole plants, portions of the plant, or fruit were ground in an electric grinder. Dried CZ (50 g), CZ flower (50 g), or CT fruit (100 g) were extracted with 1,000, 500, and 500 mL of ethanol, respectively, at 60°C under an ultrasonic condition for 2 h. For water extract preparation, dried CZ (50 g) and CT fruits (100 g) were extracted with 500 mL of water under pressure at 121°C for 1 h. The ethanol and water extracts were then filtered, and concentrates were dissolved in dimethyl sulfoxide (DMSO) or distilled water, respectively. All extract stock solutions were stored at −20°C until use.

### 2.3. DPPH and ABTS Assay

Antioxidant capacity was analyzed using the DPPH (2,2-diphenyl-1-picryl-hydrazyl-hydrate) and ABTS (2,2-azino-bis-3-ethylbenzothiazoline-6-sulfonic acid) assays. Samples were diluted appropriately with DMSO, and ascorbic acid was used as an antioxidant standard. The DPPH assay was performed as described in a previous study [11, 12]. A solution of 0.2 mM DPPH in 80% (v/v) methanol was stirred for 1 h, and the absorbance of the solution was adjusted to 0.650 ± 0.020 at 517 nm using fresh 80% (v/v) methanol. Then, 10 *μ*L of sample was mixed with 90 *μ*L of DPPH solution and incubated at 25°C for 10 min in the dark (covered with aluminum foil). Absorbance was assessed at 517 nm. For the ABTS assay, 7 mM of ABTS was mixed at a 1 : 1 ratio with 2.45 mM of potassium persulfate and stirred at 25°C for 16 h. The mixture was diluted with fresh PBS buffer until the absorbance at 734 nm reached a value of 1. Then, 10 *μ*L of sample was mixed with 90 *μ*L of the ABTS solution and incubated at 25°C for 10 min in the dark (covered with aluminum foil). Absorbance was assessed at 734 nm. Standard curves for both assays were obtained by measuring the DPPH and ABTS scavenging activities of 2, 4, 10, 20, and 140 *μ*g vitamin C/ml. All reactions were performed in triplicate. The following equation was used: DPPH or ABTS radical scavenger (%) = (1 − sample absorbance/control absorbance) × 100.

### 2.4. Cell Cultures

Mouse macrophage RAW 264.7 cells were maintained in Dulbecco's modified Eagle's medium (DMEM) supplemented with 10% (v/v) fetal bovine serum (FBS) (Hyclone, USA) and 50 *μ*g/ml gentamycin (Sigma, USA) at 37°C in a humidified 5% CO_2_ atmosphere.

### 2.5. Cell Viability Test

RAW 264.7 cells were plated to 24-well plates at 2 × 10^6^ cells/well. After 24 h, cells were treated with various concentration of extracts and incubated for a further 24 h. MTT solution was added to cells, and the plates were incubated for 1 h at 37°C. Formazan crystals were dissolved with a 1 : 1 solution of DMSO : EtOH, and absorbance at 570 nm was read on a microplate reader.

### 2.6. RT-PCR

Total RNA from RAW 264.7 cells was extracted with easy-BLUE™ (iNtRON, Korea). cDNA was synthesized from 2 *μ*g of total RNA using the reverse transcription system (Promega, USA). PCR was performed using primers against mouse iNOS (F: 5′-GCA TCC CAA GTA CGA GTG GT-3′, R: CCA TGA TGG TCA CAT TCT GC-3′), IL-6 (F: 5′-AGT TCT TCG TAG AGA ACA AC-3′, R: 5′-TTC TGG AGT ACC ATA GCT AC-3′), COX-2 (F: 5′-GCT GTA CAA GCA GTG GCA AA-3′, R: 5′-GTC TGG AGT GGG AGG CAC T-3′), and *β*-actin (F: 5′-TGG AAT CCT GTG GCA TCC ATG AAA-3′, R: 5′-TAA AAC GCA GCT CAG TAA CAG TCC G-3′). The PCR products were electrophoresed on 1% agarose gel. The gels were observed and imaged using UV imaging equipment.

### 2.7. Measurement of Intracellular Reactive Oxygen Species (ROS)

RAW 264.7 cells were treated with or without 100 *μ*g/ml of extract and stimulated with 0.1 *μ*g/ml LPS for 24 h. The cells were thereafter washed with PBS and incubated with serum-free DMEM containing 50 *μ*M DCF-DA for 30 min at 37°C. Cells were washed with cold PBS and lysed with RIPA buffer, and DCF fluorescence was detected at absorbance Ex/Em = 485/535 nm using a fluorescence microplate reader (SpectraMax i3; Molecular Devices, USA).

### 2.8. Immunofluorescence Staining

RAW 264.7 cells on cover glasses were treated with or without the indicated concentrations of extract with 0.1 *μ*g/ml LPS for 0.5 h. The cells were then fixed with 3.5% paraformaldehyde for 10 min, washed with PBS, and permeabilized with 0.5% Triton X-100 in PBS for 15 min. The slides were incubated with 0.5% BSA blocking buffer and probed with anti-p65 antibody for overnight at 4°C. After being washed with PBS, the slides were incubated with FITC-conjugated anti-rabbit antibody (Life Technologies, Eugene, OR) at room temperature for 1 h. Each cover glass was mounted on a glass slide with mounting solution containing 4′,6-diamidino-2-phenylindole (DAPI) and visualized by confocal microscopy (Carl Zeiss, Gottingen, Germany).

### 2.9. Western Blotting

RAW 264.7 cells were washed with PBS and lysed in NP-40 lysis buffer (150 mM NaCl, 50 mM TrispH 8.0, and 0.5% NP-40) containing protease inhibitors (1 *µ*g/ml aprotinin, 1 *µ*g/ml antipain, 5 *µ*g/ml leupeptin, 1 *µ*g/ml pepstatin A, and 20 *µ*g/ml phenylmethylsulfonyl fluoride) and phosphatase inhibitors (10 *µ*M NaF and 2 *µ*M Na_3_VO_4_). Cell lysates were incubated on ice for 10 min and clarified by centrifugation at 13,000 rpm for 15 min at 4°C. Lysates were boiled with SDS loading sample buffer for 5 min, and protein samples were separated in 10% SDS-PAGE, transferred to nitrocellulose blotting membranes (GE Healthcare), and probed with either anti-I*κ*B*α* (Cell Signaling, Beverly, MA) or anti-*β*-actin (Santa Cruz Biotechnology Inc., Dallas, Texas) antibodies. Signals were detected with Odyssey (Li-Cor, Lincoln, NE).

### 2.10. Statistical Analysis

Data are expressed as the mean ± standard deviation (S.D., error bars) of three independent experiments. Statistical comparison of the results was carried out using one-way analysis of variance (ANOVA) followed by Tukey's post hoc test. A *p* value <0.05 was considered statistically significant.

## 3. Results

### 3.1. Extracts from *Chrysanthemum zawadskii* Reduce LPS-Induced Inflammatory Responses

**Figure 1:**
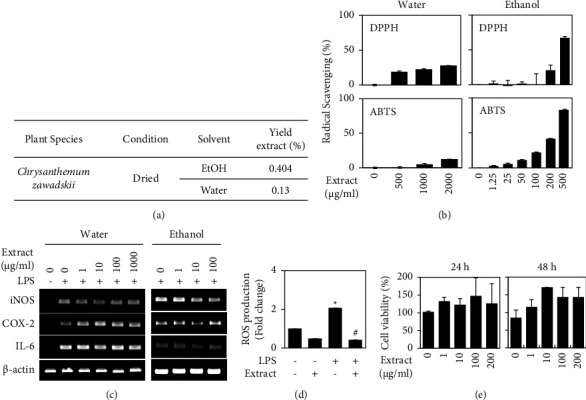
Antioxidant and anti-inflammatory effects of ethanol extracts from dried *Chrysanthemum zawadskii.* (a) Extraction yield of dried *Chrysanthemum zawadskii* (CZ) and (b) antioxidant activity was determined using the DPPH assay (top panel) or the ABTS assay (bottom panel). The indicated concentration of water (left panel) or ethanol extract (right panel) from dried CZ was mixed with assay buffer, and the free radical-scavenging ability was analyzed as described in the Materials and Methods. (c) RAW 264.7 cells were treated with the indicated amount of the extract with or without 0.1 *μ*g/ml of LPS for 24 h. The mRNA levels of iNOS, COX-2, and IL-6 were analyzed using RT-PCR. *β*-Actin was detected as a control. (d) RAW 264.7 cells were incubated with the extract (100 *μ*g/ml) in the presence or absence of 0.1 *μ*g/ml of LPS for 24 h. Intracellular ROS levels were measured by DCA-DA assay. (e) RAW 264.7 cells were treated with the indicated amount of extract for the indicated period. The MTT assay was performed, and the results are presented as the percentage of untreated control. Data were represented as mean ± standard deviation (SD). ^*∗*^*P* < 0.05 vs. control. ^#^*P* < 0.05 vs. LPS treatment.

To investigate the anti-inflammatory effects of CZ, we first extracted natural ingredients from dried whole CZ specimens comprising stem, leaf, and flower. Water and ethanol extractions produced yields of 0.13% and 0.404%, respectively (Figure 1(a)). We then tested the antioxidant potentials of the water- and ethanol-soluble extracts using the DPPH and ABTS assays, which are typical *in vitro* assays for free radical-scavenging ability. As shown in Figure 1(b), both extracts enhanced free radical scavenging in a dose-dependent manner. Under our experimental conditions, 500 *μ*g/ml of water-soluble extract did not exhibit free radical scavenging, whereas the same concentration of ethanol-soluble extract exhibited good free radical-scavenging activity (82.8% in the ABTS assay and 67.23% in the DPPH assay). Thus, ethanol-soluble extracts appeared to have a better antioxidant effect. Next, to investigate the anti-inflammatory effect of CZ, we established a cellular model by applying lipopolysaccharide (LPS) to the murine macrophage cell line, RAW 264.7. As shown in Figure 1(b), ethanol extracts showed anti-inflammatory effects. Our assays revealed that LPS (0.1 *μ*g/ml) induced the mRNA expression levels of iNOS, COX-2, and IL-6 in RAW 264.7 cells at 24 h after stimulation. The ethanol extract (100 *μ*g/ml) reduced the LPS-induced upregulations of these genes by 28%, 38%, and 30%, respectively, but such effects were not seen in cultures treated with the water extract (Figure 1(c)). The ethanol-soluble extracts were also found to reduce LPS-induced intracellular ROS production in RAW 264.7 cells (Figure 1(d)) but did not negatively affect cell viability; rather, the treatment somewhat increased the proliferation of RAW 264.7 cells (Figure 1(e)). Together, these data suggest that a whole-plant ethanol extract of CZ exhibits considerable anti-inflammatory potential.

### 3.2. The *Chrysanthemum zawadskii* Flower Endows Anti-Inflammatory Effects

**Figure 2:**
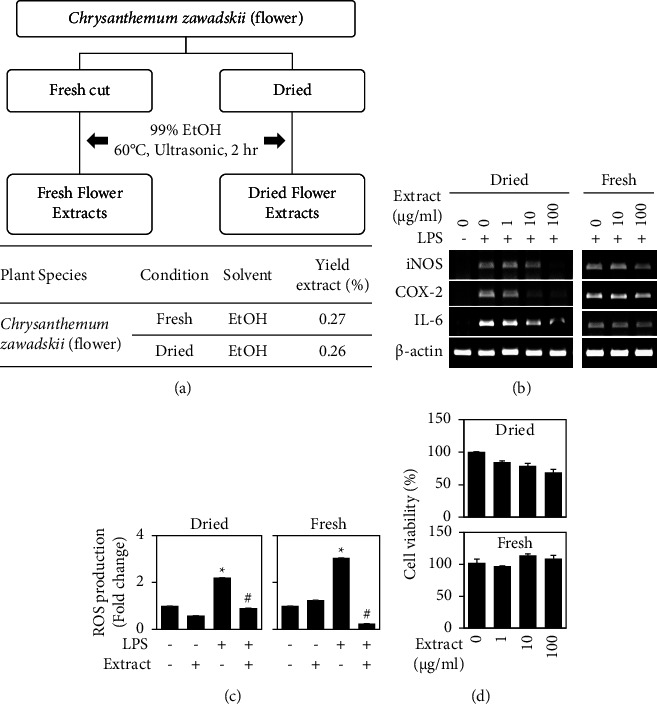
Anti-inflammatory effects of ethanol extracts from *Chrysanthemum zawadskii* flowers. (a) Fresh and dried flowers of CZ were extracted using 99% ethanol (top panel) and percentage of yield extract (bottom panel) as indicated. (b) RAW 264.7 cells were treated with the indicated amount of extract with or without 0.1 *μ*g/ml of LPS for 24 h. The expression levels of mRNAs were analyzed using RT-PCR. *β*-Actin was detected as a control. (c) RAW 264.7 cells were incubated with extract in the presence or absence of 0.1 *μ*g/ml of LPS for 24 h. Intracellular ROS levels were measured by DCA-DA assay. (d) RAW 264.7 cells were treated with the indicated amount of extract for 24 h, and MTT assay was performed. Results are presented as a percentage of the untreated control. Data were represented as mean ± standard deviation (SD). ^*∗*^*P* < 0.05 vs. control. ^#^*P* < 0.05 vs. LPS treatment.

**Figure 3:**
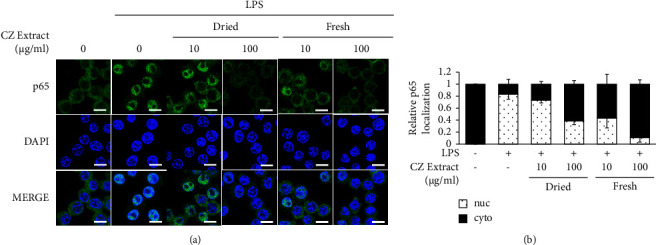
Anti-inflammatory signaling effects of ethanol extracts from *Chrysanthemum zawadskii* flowers. RAW 264.7 cells were treated with the indicated amounts of ethanol extract with or without 0.1 *μ*g/ml of LPS for 30 min. (a) The nuclear translocation of NF-*κ*B subunit p65 was assessed using immunofluorescence. (b) Nuclei were counterstained with DAPI, and positive staining for p65 was quantified and graphed. Scale bar, 10 *μ*m.

Assuming that the flower was the more potent source of active ingredients, we further investigated the anti-inflammatory effects of ethanol extracts from the CZ flower (Figure 2). We obtained the natural ingredients at a yield of around 0.27% for both fresh and dried flowers (Figure 2(a)). As expected, both extracts reduced the LPS-induced upregulations of the transcripts encoding iNOS, COX-2, and IL-6 (Figure 2(b)) and production of intracellular ROS (Figure 2(c)) in RAW 264.7 cells. In particular, 100 *μ*g/ml of the ethanol extract from dried flowers reduced the LPS-induced upregulations of the transcripts for iNOS and COX-2, whereas the extract from fresh flowers mildly decreased these parameters (Figure 2(b)), suggesting anti-inflammatory activities of ethanol extracts from both dried and flesh flowers. Our MTT data further revealed that ethanol extracts from fresh flowers showed no cytotoxicity up to 100 *μ*g/ml in RAW 264.7 cells, whereas ethanol extracts from dried flowers exhibited some slight cytotoxicity against RAW 264.7 cells at this concentration (Figure 2(d)), supporting our contention that CZ flower extracts exhibit anti-inflammatory effects with low potential for side effects. Since LPS is known to stimulate inflammatory gene expression through the activation of nuclear factor-kappa B (NF-*κ*B) [13, 14], we further examined the effect of the extract on NF-*κ*B (Figure 3). Immunofluorescence staining showed that both dried and fresh flower extracts dose-dependently reduced the LPS-induced nuclear translocation of NF-*κ*B subunit p65 (Figure 3). Together, these results indicate that ethanol extracts from both dried and fresh flowers have anti-inflammatory activities.

### 3.3. *Cudrania tricuspidata* Fruit Extract Reduces LPS-Induced Free Radical Production and Upregulation of Proinflammatory Gene Expression

**Figure 4:**
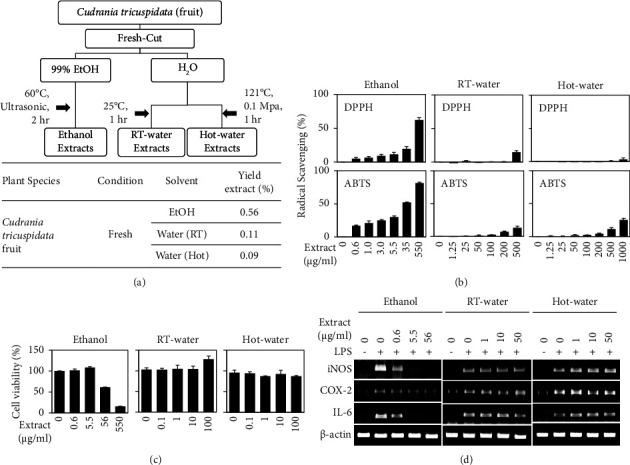
Anti-inflammatory effects of *Cudrania tricuspidata* fruit extracts. (a) *Cudrania tricuspidata* (CT) fruit was extracted using 99% ethanol, room temperature water or hot water (top panel). Extraction yield of CT fruit (bottom panel). (b) The indicated concentration of extract from CT fruit was mixed with assay buffer and analyzed for the free radical-scavenging ability. (c) RAW 264.7 cells were treated with the indicated amount of extract for 24 h, and the MTT assay was performed. Results are presented as a percentage of the untreated control and represent the mean ± SEM. (d) RAW 264.7 cells were treated with the indicated amount of extract with or without 0.1 *μ*g/ml of LPS for 24 h. The expression levels of mRNAs were analyzed using RT-PCR. *β*-Actin was detected as a control. Data were represented as mean ± standard deviation (SD). ^*∗*^*P* < 0.05 vs. control.

We then searched for a natural product that might potentiate the anti-inflammatory effects of CZ. CT has been used in traditional fermented jams, juices, and alcoholic beverages in Korea. In terms of active ingredients, the fruit of CT has been shown to contain prenylated isoflavonoids, benylated flavonoids, and 5,7,3′,4′-tetrahydroxy-6,8-diprenylisoflavone [15]. Since CT is known to exhibit anti-inflammatory effects [16] and protect cells against oxidative stress-induced neurotoxicity [17], we examined the anti-inflammatory effect of CT (Figure 4). We extracted natural ingredients from CT fruit with either water or ethanol (Figure 4(a)). Ethanol extraction showed a high yield of 0.6%, whereas room temperature water and hot water both showed low yields of ∼0.1% (Figure 4(a)). Similar to our findings with CZ, the ethanol extracts showed much higher antioxidant and anti-inflammatory activities (Figures 4(b) and 4(d)). At 550 *μ*g/ml, the ethanol extract showed free radical-scavenging activity (81.0% in the ABTS assay and 62.0% in the DPPH assay) similar to that obtained with the CZ extract; however, only very weak activities were seen for both water-soluble extracts (Figure 4(b)). The ethanol extract showed no cytotoxicity up to a dose of 5.5 *μ*g/ml (Figure 4(c)). At this nontoxic concentration, the ethanol extract completely abolished the LPS-induced upregulations of iNOS and IL-6 gene expression (Figure 4(d)). These data suggest that ethanol extracts of CT fruit have anti-inflammatory effects.

### 3.4. Synergistic Anti-Inflammatory Effects of Ethanol Extracts from *Chrysanthemum zawadskii* Flower and *Cudrania tricuspidata* Fruit

**Figure 5:**
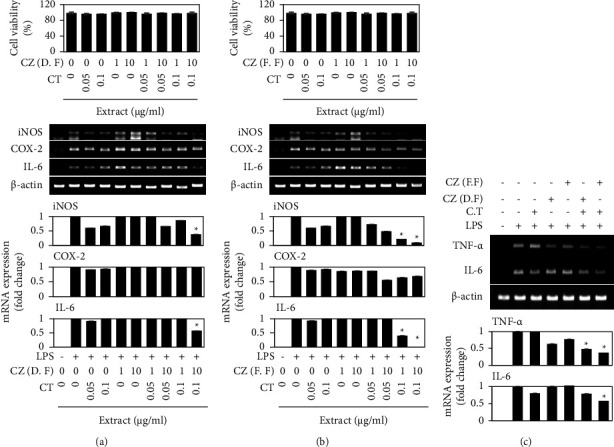
Synergistic anti-inflammatory effect of extracts from *Chrysanthemum zawadskii* flower and *Cudrania tricuspidata* fruit. RAW 264.7 cells were treated with each extract alone or cotreated with both extracts. The ethanol extracts of CZ were from either dried flower (a) or fresh flower (b). Cell viability was determined using the MTT assay (top panel). The mRNA levels of iNOS, COX-2, and IL-6 were analyzed using RT-PCR (bottom panel). (c) RAW 264.7 cells were treated with each extract alone or cotreated with both extracts as indicated. The mRNA levels of TNF-*α* and IL-6 were analyzed using RT-PCR. Data were represented as mean ± standard deviation (SD). ^*∗*^*P* < 0.05 vs. LPS treatment.

**Figure 6:**
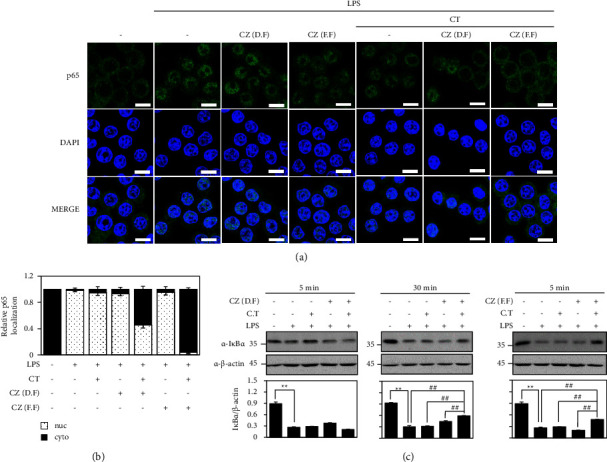
Synergistic anti-inflammatory signaling effect of extracts from *Chrysanthemum zawadskii* flower and *Cudrania tricuspidata* fruit. RAW 264.7 cells were treated with 0.1 *μ*g/ml of CT fruit extract and 5 *μ*g/ml of CZ flower extracts alone or cotreated with both extracts. Cells were stimulated with 0.1 *μ*g/ml of LPS for 30 min (a, b) or the indicate time (c). (a) The nuclear translocation of NF-*κ*B subunit p65 was assessed using immunofluorescence. (b) Nuclei were counterstained with DAPI, and positive staining for *p*65 was quantified and graphed. Scale bar, 10 *μ*m. (c) The expression levels of protein were analyzed by Western blot analysis using anti-I*κ*B*α* antibodies. *β*-Actin was detected as a control. The expression of I*κ*B*α* was quantified and presented graphically. ^*∗∗*^*P* < 0.01 vs. control. ^##^*P* < 0.01 vs. LPS treatment.

Finally, we examined the combinatorial anti-inflammatory effect of the ethanol extracts from CZ flower and CT fruit (Figure 5). Toward this end, we combined ethanol extracts from CZ flower and CT fruit at concentrations that did not exert cytotoxicity under our experimental condition, as verified by MTT assays of RAW 264.7 cells treated with either extract alone or cotreated with both extracts together (Figure 5(a)). Interestingly, although monotreatment with 10 *μ*g/ml of ethanol extract from dried flowers of CZ or 0.1 *μ*g/ml of ethanol extract from CT fruits failed to alter the LPS-induced upregulations of iNOS, COX-2, and IL-6, cotreatment of these concentrations together significantly reduced the LPS-induced upregulations of iNOS and IL-6 (Figure 5(a)). Furthermore, cotreatment of ethanol extracts from fresh flowers of CZ and CT fruit showed better anti-inflammatory effects, with only 1.0 *μ*g/ml of each extract needed to significantly reduce the LPS-induced upregulations of iNOS and IL-6 (Figure 5(b)). Consequently, cotreatment with these extracts significantly reduced the LPS-induced upregulations of TNF-*α*, another important inflammatory factor, of RAW 264.7 cells (Figure 5(c)). In addition, this cotreatment with low amounts of each extract efficiently restored the LPS-induced nuclear localization of p65 (Figures 6(a) and 6(b)). In addition, this cotreatment with low amounts of each extract efficiently restored the LPS-induced nuclear localization of p65 (Figures 6(a) and 6(b)) and degradation of I*κ*B*α* (Figure 6(c)). Interestingly, cotreatment of these extracts containing extracts from dried CZ flowers showed a synergistic restoration of I*κ*B*α* levels after 30 min, but this effect was observed with the extract from fresh CZ flowers of CZ after 5 min (Figure 6(c)), supporting better anti-inflammatory effects of fresh flowers. Taken together, these data indicate that ethanol extracts from CZ flowers and CT fruits have a synergistic anti-inflammatory effect.

## 4. Discussion

CZ has been used as an herbal medicine in Korea for various diseases, such as gastroenteric diseases, bladder-related diseases, and uterine diseases including menstrual disorders and infertility [18, 19]. The beneficial effects are thought to be associated with anti-inflammatory effects. Consistent with previous studies, our data confirmed that CZ was effective as an antioxidant and anti-inflammatory agents. In particular, ethanol extracts from fresh flowers showed better effectiveness than those of dried flowers or dried whole CZ (Figures 1 and 2). Interestingly, the CZ flower ethanol extracts showed better anti-inflammatory activity than the whole dried CZ at the dose of 100 *μ*g/ml (Figures 1 and 2).

CT fruit has also been used as an herbal remedy in Korea. A previous study showed that an active compound isolated from CT fruit exerted antiatherosclerotic and neuroprotective activities [20]. CT extracts were found to inhibit the LPS-induced upregulations of iNOS expression, COX-2 expression, and NO production [21, 22] and to suppress *Dermatophagoides farinae*-induced atopic dermatitis [23]. Consistently, the ethanol extracts of CT fruit showed anti-inflammatory activities similar to those of CZ flowers (Figure 4). When we tested various combinations of the two extracts, 10 *μ*g/ml of ethanol extract from fresh flowers of CZ and 0.1 *μ*g/ml of ethanol extract from CT fruits completely inhibited the LPS-induced upregulations of iNOS and IL-6 (Figure 5(b)). Since the combination of ethanol extract from fresh flowers of CZ and ethanol extract from fruits of CT exerted synergistic inhibitory effects on multiple proinflammatory biomarkers, including iNOS and IL-6 (Figure 5), we investigated the underlying molecular mechanisms. The NF-*κ*B signaling pathway is known to be essential for regulating inflammation [13, 14]. Consistent with this, our results demonstrated that ethanol extracts of both fresh flowers of CZ decreased the LPS-stimulated nuclear localization of p65 (Figure 3), and combination treatment with extracts from both fresh flowers of CZ and fruits of CT showed more potent inhibition than either monotreatment, at lower doses (Figure 6). Whereas 100 *μ*g/ml of the ethanol extract from dried flower of CZ significantly reduced the ability of LPS to upregulate the mRNAs encoding iNOS and IL-6 (Figure 2) and decreased the LPS-induced nuclear localization of p65 (Figure 3), cotreatment of ethanol extracts from fresh flowers of CZ and fruits of CT showed better anti-inflammatory effects, with only 1.0 *μ*g/ml of each extract needed to significantly reduce the LPS-induced upregulations of iNOS and IL-6 (Figure 5). Here, we show that cotreatment with low doses of each extract reduced the LPS-induced nuclear localization of p65 (Figure 6). These findings suggest that the observed synergistic anti-inflammatory effects occur via inhibition of the NF-*κ*B signaling pathway.

In summary, since the corresponding monotreatments had only mild effects, we conclude that these extracts together have a synergistic anti-inflammatory effect. This synergism strongly suggests that the cotreatment of these compounds could replace the natural material whose use is limited due to its toxicity despite its anti-inflammatory properties. Also, given that these plants have long been used as folk remedies, they are expected to have high safety when coapplied to treat inflammation and inflammation-related diseases.

## Data Availability

The data used to support the findings of this study are available from the corresponding author upon request.
